# Exploring the Impact of Chirality of Synthetic Cannabinoids and Cathinones: A Systematic Review on Enantioresolution Methods and Enantioselectivity Studies

**DOI:** 10.3390/ijms26136471

**Published:** 2025-07-04

**Authors:** Ana Sofia Almeida, Rita M. G. Santos, Paula Guedes de Pinho, Fernando Remião, Carla Fernandes

**Affiliations:** 1 Laboratório de Química Orgânica e Farmacêutica, Departamento de Ciências Químicas, Faculdade de Farmácia, Universidade do Porto, Rua Jorge Viterbo Ferreira, 228, 4050-313 Porto, Portugal; anasofiaalmeida1998@gmail.com (A.S.A.); anaritasantos7890@gmail.com (R.M.G.S.); 2 Centro Interdisciplinar de Investigação Marinha e Ambiental (CIIMAR), Universidade do Porto, Terminal de Cruzeiros do Porto de Leixões, Avenida General Norton de Matos, s/n, 4450-208 Matosinhos, Portugal; 3 UCIBIO-Applied Molecular Biosciences Unit, Laboratory of Toxicology, Department of Biological Sciences, Faculty of Pharmacy, University of Porto, Rua Jorge Viterbo Ferreira, 228, 4050-313 Porto, Portugal; pguedes@ff.up.pt; 4 Associate Laboratory i4HB—Institute for Health and Bioeconomy, University of Porto, Rua Jorge Viterbo Ferreira, 228, 4050-313 Porto, Portugal

**Keywords:** enantioselectivity, enantioresolution, new psychoactive substances, synthetic cannabinoids, synthetic cathinones

## Abstract

New psychoactive substances (NPSs) are emerging narcotics or psychotropics that pose a public health risk. The most commonly reported NPSs are synthetic cannabinoids and synthetic cathinones. Synthetic cannabinoids mimic the effects of Δ9-tetrahydrocannabinol (Δ9-THC), often with greater potency, while synthetic cathinones act as stimulants, frequently serving as cheaper alternatives to amphetamines, 3,4-methylenedioxymethamphetamine (MDMA) and cocaine. While some synthetic cannabinoids exhibit chirality depending on their synthesis precursors, synthetic cathinones are intrinsically chiral. Biotargets can recognize and differentiate between enantiomers, leading to distinct biological responses (enantioselectivity). Understanding these differences is crucial; therefore, the development of enantioresolution methods to assess the biological and toxicological effects of enantiomer is necessary. This work systematically compiles enantioselectivity studies and enantioresolution methods of synthetic cannabinoids and synthetic cathinones, following PRISMA guidelines. The main aim of this review is to explore the impact of chirality on these NPSs, improving our understanding of their toxicological behavior and evaluating advances in analytical techniques for their enantioseparation. Key examples from both groups are presented. This review highlights the importance of continuing research in this field, as demonstrated by the differing properties of synthetic cannabinoid and synthetic cathinone enantiomers, which are closely linked to variations in biological and toxicological outcomes.

## 1. Introduction

The European Union Drugs Agency (EUDA) defines a new psychoactive substance (NPS) as “a new narcotic or psychotropic in pure form or in preparation, that is not controlled by the United Nations drug conventions, but which may pose a public health threat comparable to that posed by substances listed in these conventions” [1]. NPSs are commonly known as synthetic or designer drugs. They represent an extensive and diverse group of substances, often analogs of already legally controlled drugs [2]. NPSs are generally marketed as “bath salts” or “plant food”, accompanied by the disclaimer “not for human consumption” to bypass the country’s drug legislation [3].

**Figure 1:**
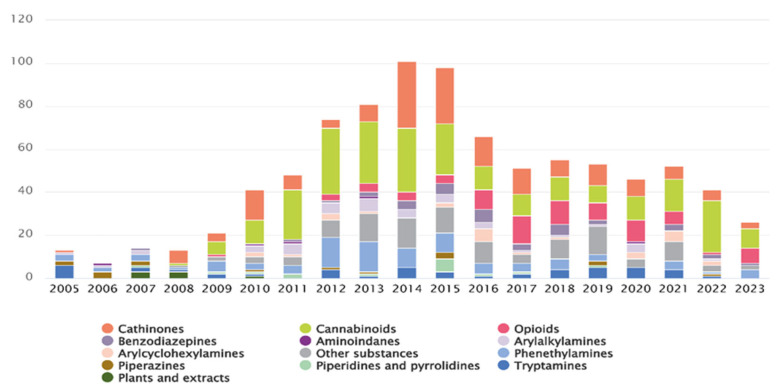
Number and categories of new psychoactive substances reported for the first time from 2005 to 2023 [5].

As of 2021, the EUDA was monitoring 880 NPSs, including 370 first reported in 2020 [4]. By the end of 2023, this number had risen to approximately 950, with 26 newly reported in Europe that year [5]. The rapid emergence of these new substances is unprecedented and alarming, with estimates suggesting that, at its peak, a new NPS appeared every week [2]. Figure 1 illustrates the number and categories of NPSs first reported between 2005 and 2023 [5].

The popularity of these compounds has grown mainly due to their psychoactive effects, namely their stimulatory, hallucinogenic and depressant effects, and because they are easily available online. However, many NPSs remain unstudied, and data on their toxicological and pharmacological properties is limited. Additionally, the composition of NPSs purchased online may differ from what is stated on the packaging, leading users to unknowingly consume unlisted substances. These factors emphasize the significant public health risks associated with NPS use [3,6]. According to EUDA, NPSs are classified into many categories, based on their chemical structure (Figure 1) [5]. Among these, synthetic cannabinoids and synthetic cathinones are the most commonly reported, accounting for more than two-thirds of all available NPSs [7].

**Figure 2:**
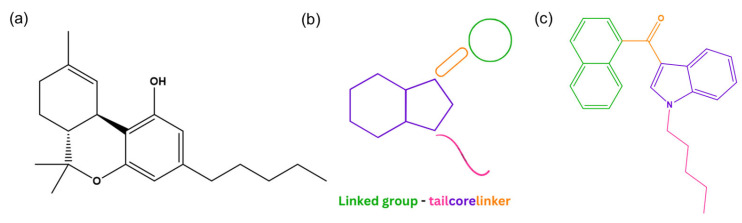
Structure of Δ^9^-THC (**a**), structural model of synthetic cannabinoids (**b**), and structure of JWH-018 (**c**). (ChemDraw^®^ Professional 18.0).

Synthetic cannabinoids first emerged in the 1960s and the 1970s when researchers began studying the endocannabinoid system’s role in biological functions [8,9,10]. They are a chemically and structurally diverse group of NPSs designed to target the endocannabinoid system. These substances exhibit a higher affinity for cannabinoid receptors (CBRs) than Δ^9^-tetrahydrocannabinol (Δ^9^-THC, Figure 2a), the main psychoactive compound in cannabis, resulting in Δ^9^-THC-like effects that can be more potent and vary in duration (either longer or shorter depending on the specific substance) [10,11]. To classify these substances, the EUDA proposed a model that identifies the names of the key structural components of each molecule: a “core”, a “linker”, a “linked group” and a “tail” (Figure 2b) [12]. Depending on the chemical nature of the linked group, some synthetic cannabinoids can be chiral, meaning they exist as two enantiomers [13]. Since the detection of JWH-018 (Figure 2c) in the drug market in 2008 [8,14], synthetic cannabinoids have dominated the NPS market, particularly between 2008 and 2013, as well as in recent years (2020–2023) [15].

**Figure 3:**
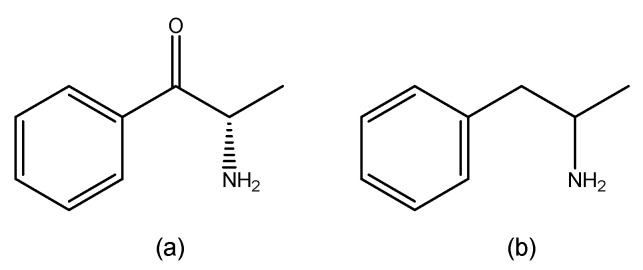
Structures of cathinone (**a**) and amphetamine (**b**) (ChemDraw^®^ Professional 18.0).

On the other hand, synthetic cathinones are a group of compounds derived from cathinone (Figure 3a), a natural alkaloid found in *Catha edulis* leaves, commonly known as khat. Structurally, cathinone resembles amphetamine (Figure 3b) [7], with the key difference being the presence of a ketone group at the β-position of the amino alkyl chain attached to the phenyl ring [16].

This chemically diverse group of NPSs is widely abused for its psychostimulant effects, similar to those of cocaine or 3,4-methylenedioxymethamphetamine (MDMA) [17,18]. Their simple molecular structure and high variability facilitate the development of new analogs with unknown pharmacological and toxicological properties. This allows them to evade legal restrictions, making them more dangerous, as reports of fatal intoxications continue to rise [19]. All synthetic cathinones are chiral molecules. Since biotargets are intrinsically chiral, they can recognize and differentiate between enantiomers, leading to distinct biological responses (enantioselectivity). As a result, each enantiomer may exhibit different biological and/or toxicological properties [20]; therefore, enantioresolution methods are essential for assessing their individual impacts [21].

The relationship between chirality and biological activity has gained increasing importance since Louis Pasteur’s [22] first observation of biological enantioselectivity and the tragic thalidomide incident [23,24]. Chirality can now be considered one of the major topics in many research fields, including the medical and toxicological sciences [20,25,26]. Furthermore, in the past few decades, numerous analytical and preparative methods for enantioresolution have been developed to facilitate the analysis and isolation of single enantiomers [27,28]. This work compiles enantioselectivity and enantioseparation studies on both synthetic cannabinoids and synthetic cathinones. To the best of our knowledge, no other review exists for synthetic cannabinoids. On the other hand, reviews on synthetic cathinones (published in 2018 and 2022) indicate a growing research focus in this area [7,29]. Therefore, an update on recent developments, particularly from the past three years, is necessary to track this progression. This review aims to explore the impact of chirality in both groups of NPSs by examining reports on the differing effects of their enantiomers across various biological systems and evaluating advances in analytical techniques for their enantioseparation.

The key novelty of this review lies in it being the first to analyze the impact of chirality on synthetic cannabinoids, thereby enhancing our understanding of their toxicological behavior, as stereochemical features are often closely linked to variations in biological and toxicological outcomes.

## 2. Literature Research Methodology (PRISMA Guidelines)

This research followed the Preferred Reporting Items for Systematic Reviews and Meta-Analyses (PRISMA) guidelines [30]. Research on the literature was conducted in the PubMed and SCOPUS databases to identify studies on enantioselectivity and enantioseparation methods for synthetic cannabinoids. The search used the following keywords: “Synthetic cannabinoids AND chiral OR enantioresolution OR enantioseparation OR enantioselectivity OR enantiomer OR stereochemical OR stereochemistry”. Study selection took place in February 2025, with the earliest identified study dating back to 1989. Only English-language papers were considered. A total of 156 records were identified and screened for duplicates. In the first screening phase, 78 records were assessed by title and abstract to exclude irrelevant studies and review papers. In the second phase, a full-text review of 65 records was conducted, further eliminating non-relevant studies. Additionally, studies identified through other methods, such as citation searching, were included. The final selected records are categorized into enantioselectivity studies, enantioseparation studies or both.

Similarly, to identify relevant studies on enantioselectivity and enantioseparation methods for synthetic cathinones, a literature search was conducted in the PubMed and SCOPUS databases using the following keywords: “Synthetic cathinones AND chiral OR enantioresolution OR enantioseparation OR enantioselectivity OR enantiomer OR stereochemical OR stereochemistry”. The study selection took place in February 2025. Since this research is an update of previous review papers [7,29], only studies published between 2022 and February 2025 were included. Only English-language papers were considered. A total of 94 records were identified and screened for duplicates. In the first screening phase, 57 records were assessed by title and abstract to exclude irrelevant studies and review papers. In the second phase, a full-text review of 48 records was conducted, with further exclusions of non-relevant studies. Additionally, studies identified through other methods, such as citation searching, were included. The final selected records were categorized into enantioselectivity studies, enantioseparation studies, or both.

Screening at both the title/abstract and full-text stages and data collection were initially performed by two reviewers. All screening and data collection outcomes were subsequently discussed and reviewed by additional reviewers. No automation tools were applied. The collected data were critically and impartially analyzed in accordance with PRISMA guidelines. Most of the authors have previous experience in PRISMA analysis [31,32,33,34], along with topics of research such as NPSs [35,36,37], enantioselectivity studies [38,39,40] and chiral separations [17,41,42].

This review focused on enantioselectivity and enantioseparation studies of synthetic cathinones and synthetic cannabinoids. Therefore, the inclusion criteria were original research articles on enantioselectivity studies (biological/toxicological assays) and enantioseparation methods using synthetic cannabinoids or synthetic cathinones. The exclusion criteria included reviews, theoretical/computational studies, articles focusing on non-chiral molecules and studies lacking either enantioselectivity or enantioresolutiona. For enantioselectivity studies, the primary outcomes were the distinct effects of enantiomers in various biological systems. For enantioseparation methods, relevant experimental conditions such as analytes, samples, methods, and analytical conditions were noted. If studies reported inconsistent outcomes or incomplete outcomes, data were selected based on their relevance to the review’s objective and consistency with other results.

Given the heterogeneity in study designs and outcome metrics, we performed a descriptive synthesis. To summarize the enantioselectivity results for both NPS groups, the main findings from each study were highlighted. For synthetic cannabinoids, studies on the same class of compounds were grouped together. Additionally, the structure of some compounds was generated using ChemDraw 18.0.

For the enantioseparation studies, a table was created to present the analytes, samples, methods, and analytical conditions relevant for each approach. Bar charts illustrating the distribution of analytical techniques, chiral selectors, and detection methods used in the enantioseparation studies of synthetic cathinones and cannabinoids were created using GraphPad Prism 9. No statistical or meta-analysis was conducted on the data.

The main findings of the enantioseparation methods were analyzed and discussed according to various topics: technique, chiral selector, detection method, and sample type. For synthetic cannabinoids, the most prevalent classes of cannabinoids in the studies were also discussed. For synthetic cathinones, a comparison with previous reviews was made to provide a more comprehensive analysis of enantioseparation methods. Additionally, a comparison between the enantioseparation methods for synthetic cannabinoids and synthetic cathinones was conducted, with representative examples from the selected studies described. No meta-analysis was performed.

Although a formal risk of bias assessment was not conducted, we acknowledge the potential for bias in the reviewed studies due to variations in study design, sample selection, data collection methods, and statistical analyses. Furthermore, the possibility of publication bias, where studies with significant or positive results are more likely to be published, is also recognized as a limitation.

To minimize potential biases, several steps were taken. The literature search was conducted using multiple databases (PubMed and SCOPUS), and citation searches were included to ensure comprehensive coverage of the relevant studies. Only original research articles were considered, while review articles were excluded to avoid duplication of findings.

## 3. Results and Discussion

### 3.1. Synthetic Cannabinoids

#### 3.1.1. PRISMA Search

**Figure 4:**
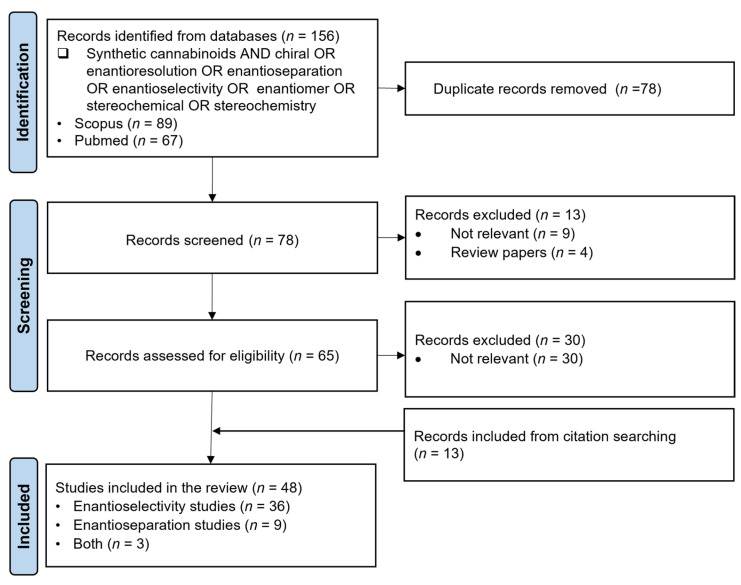
Flow diagram of the literature search based on PRISMA guidelines (*n* = number of scientific articles; time frame: 1989–February 2025; database: SCOPUS and Pubmed).

The PRISMA approach was conducted according to the flowchart presented in Figure 4. In the screening phase, some articles that seemed to meet the inclusion criteria had to be excluded since they were not relevant for the aims of this review. For instance, Huffman et al. [43] performed a synthesis of both enantiomers of nabilone. This article appeared in our search through the keywords “synthetic cannabinoids AND enantiomer”; however, it was out of the scope of this review since it was not an enantioselectivity or enantioseparation study. Moreover, Feinstein et al. [44] evaluated the effects of vitamin K1 treatment on plasma concentrations after poisoning with long-acting anticoagulant rodenticide enantiomers contaminated with synthetic cannabinoids. Although this is an enantioselectivity study and related to synthetic cannabinoids, the focus of the study is on the enantiomers of the long-acting anticoagulant rodenticides and not the enantiomers of synthetic cannabinoids; therefore, this article was excluded from the research. In the end, 48 scientific articles were selected for further discussion, 36 corresponding to enantioselectivity studies, 9 to enantioseparation studies and 3 including both enantioselectivity and enantioseparation studies.

#### 3.1.2. Enantioseparation

Although minor variations in the chiral profiles of synthetic cannabinoids have been reported, the limited number of tested samples has hindered the identification of discriminatory differences between synthesis supplies and/or final products. The rapid emergence of new CBR agonists underscore the need for quick adaptation of enantioseparation methods. Understanding enantioseparation mechanisms enables this adaptation. Reliable separation methods allow for better pharmacological and analytical assessment [13]. Therefore, chiral profiling and enantioseparation are crucial for assessing enantioselectivity [7,45] and more accurately evaluating risks to develop harm reduction strategies [13].

To our knowledge, no review has been published on the techniques developed over the years for the chiral separation of synthetic cannabinoids. This information is compiled in Table 1.

**Figure 5:**
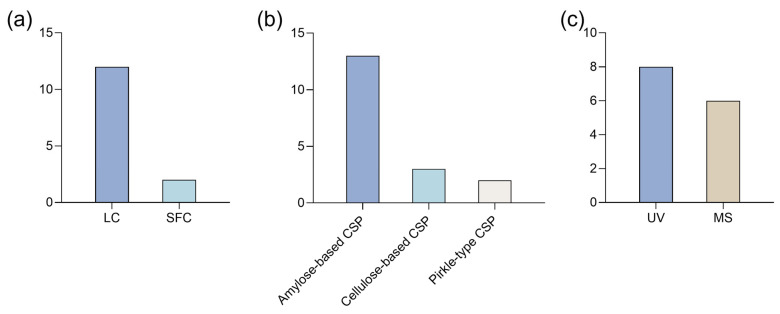
Bar charts regarding (**a**) type of technique, (**b**) chiral stationary phases (CSPs) and (**c**) detection methods used for enantioseparation of synthetic cannabinoids. LC: liquid chromatography; MS: mass spectrometry; SFC: super-critical fluid chromatography; UV: ultra-violet.

Liquid chromatography (LC), particularly high-performance liquid chromatography (HPLC), has been the predominant method for the enantioseparation of synthetic cannabinoids (Figure 5a). LC is now a standard technique for enantiomeric separations [57], offering a wide variety of chiral stationary phases (CSPs) [58] and compatibility with various detection methods, including fluorescence, ultra-violet (UV), and mass spectrometry (MS) [57].

Beyond LC techniques, two studies have explored super critical fluid chromatography (SFC), a hybrid of gas chromatography (GC) and LC that enables faster separations. However, its complexity and high cost present limitations [57]. For instance, Breitenback et al. [51] investigated the potential of an ultra-high performance SFC (UHPSFC) method for analyzing synthetic cannabinoids in samples of seized drugs. This technique demonstrated high precision and strong resolving power in separating positional isomers and diastereomers of synthetic cannabinoids [51].

According to our research, other techniques that are often applied in enantioseparation studies such as GC and capillary electrophoresis (CE) were not selected for the enantioseparation of synthetic cannabinoids.

All the studies used CSPs, indicating a preference for direct methods. The success of efficient enantioseparation largely depends on the chiral discriminative ability of CSP [59]. Polysaccharide-based CSPs were the most used, with amylose derivatives being the predominant one (Figure 5b). Indeed, polysaccharide-based CSPs are widely regarded as the most effective and commonly used CSPs for enantioseparation, both in analytical and preparative contexts [60]. This widespread use can be attributed to several factors, including the strong chiral recognition capabilities of polysaccharide derivatives and their natural abundance. For example, Antonides et al. [13] performed enantioseparation of four indazole-3-carboxamide synthetic cannabinoids using polysaccharide-based CSPs. After testing several columns, they found that Lux^®^ Amylose-1 provided the highest selectivity for compounds with a terminal methyl ester moiety, while Lux^®^ i-Cellulose-5 was more effective for those with a terminal amide moiety [13].

Only one study employed a different class of CSPs, specifically Pirkle-type CSPs. This class of CSPs, in which the chiral selector is a small molecule covalently bound to the chromatographic support via a spacer, is also broadly applied for enantioseparations [61]. Varfaj et al. [46] used the (*R*,*R*)-Whelk-O^®^ 1 and (*S*,*S*)-Whelk-O^®^ 1 columns to achieve the separation of enantiomers of synthetic cannabinoids found in seized samples.

Another key factor in the enantioseparation process is the mobile phase, which significantly affects the retention enantiomers, enantioselectivity, and resolution [62]. Both Pirkle-type and polysaccharide-based CSPs can be employed in normal-phase, reversed-phase, polar organic, and polar ionic conditions due to their compatibility with a wide range of solvents [58]. Although these CSPs support various elution modes, as shown in Table 1, normal-phase and reversed-phase conditions were the most commonly used for the enantioseparation of this class of NPSs. In particular, for normal-phase separations, mixtures of hexane and 2-propanol proved to be the most effective in achieving enantioseparation. For the reversed-phase mode, mobile phases typically consisted of water and acetonitrile, with or without an ionization suppressor such as formic acid.

Detection techniques for synthetic cannabinoids vary, with MS and UV detection being the most commonly used (Figure 5c). MS detection offers higher sensitivity and specificity [63], which makes it especially useful to identify enantiomers in complex samples, such as herbal material [13,49,52] and biological matrices like urine [53]. In some cases, both detection methods have been combined. For example, Antonides et al. [13] initially applied an HPLC method with a photodiode array (PDA) detector to test the enantioseparation of several synthetic cannabinoids using polysaccharide-based CSPs. Then, they conducted further analysis using HPLC coupled with PDA and quadrupole time of flight MS (HPLC-PDA-MS/MS) to determine the enantiopurity of the samples. The results revealed that the (*S*)-enantiomer predominated in all samples [13].

The carboxamide moiety and the JWH family of compounds were the most commonly studied cannabinoids for enantioseparation. While most analyzed samples consisted of herbal material or powders, Patton et al. [53] developed a chiral approach using LC-MS/MS to investigate the chiral metabolites of JWH-018 and AM2201 in human urine. Their aim was to evaluate enantiospecific excretion patterns and gain a better understanding of the pharmacokinetic properties of these two synthetic cannabinoids.

Although this review focuses on the enantioseparation of racemates, referred to as the “racemic approach”, to obtain individual enantiomers, an alternative method, known as the “chiral approach”, can also be used. This approach involves enantioselective synthesis to directly produce the enantiomer [64,65] and has been reported in some enantioselectivity studies with synthetic cannabinoids [50,66,67]. Nonetheless, even after enantioselective synthesis, enantioseparation methods remain valuable for assessing the enantiomeric purity of the compounds. For example, Doi et al. [50] synthesized enantiomers of several carboxamide-type synthetic cannabinoids to evaluate their activities as CB1R/CB2Rr agonists and evaluated their enantiomeric purity using an LC-MS method.

#### 3.1.3. Enantioselectivity Studies

Synthetic cannabinoids are widely studied [8,10], and several reports on enantioselectivity for specific groups can be found.

**Figure 6:**
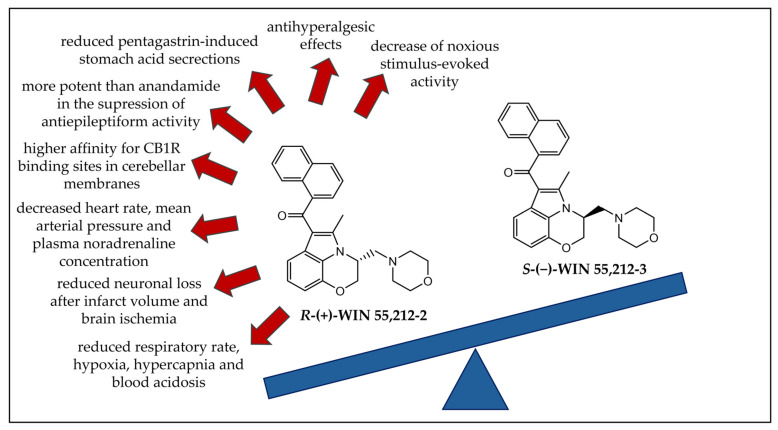
Chemical structures of *R*-(+)-WIN 55,212-2 and *S*-(−)-WIN 55,212-3 and their enantioselective effects observed in rats. CB1R: cannabinoid receptor 1.

Aminoalkylindoles are the most prevalent class of synthetic cannabinoids found in herbal products [68]. Enantioselectivity studies are crucial for understanding the duration of effects caused by this widely consumed class of substances. The aminoalkylindole derivative WIN 55,212-2 is a potent CBR agonist [69]. Numerous in vivo and in vitro studies have been conducted considering its enantioselective effects on biological activity [70,71,72,73,74,75,76,77,78,79,80,81,82,83,84,85,86,87]. The chemical structures of *R*-(+)-WIN 55,212-2 and *S*-(−)-WIN 55,212-3 are represented in Figure 6, summarizing and emphasizing the main enantioselective effects observed in rats in in vivo studies [70,71,74,75,76,81,82,83,84,85].

All the effects reported were observed only for *R*-(+)-WIN 55,212-2, while the enantiomer *S*-(−)-WIN 55,212-3 showed little or no effect. Since *S*-(−)-WIN 55,212-3 displays no pharmacological activity, these results suggest that some effects might be associated with CBRs.

Apart from these studies using rats as the in vivo model, Rawls et al. [86] performed an in vivo study using planarian to compare the effects of *R*-(+)-WIN 55,212-2 and *S*-(−)-WIN 55,212-3. The results showed that planarians display abstinence-induced withdrawal after exposure to *R*-(+)-WIN 55,212-2, but not after exposure to *S*-(−)-WIN 55,212-3.

Several in vitro studies were also reported for this compound, the results of which are summarized in Table 2 [72,73,77,78,79,80,87].

Similarly to in vivo studies, in vitro studies showed stereospecificity for *R*-(+)-WIN 55,212-2. Nonetheless, Price et al. [78] and Scholl et al. [80] reported in vitro studies where no enantioselectivity was observed for the enantiomers.

**Figure 7:**
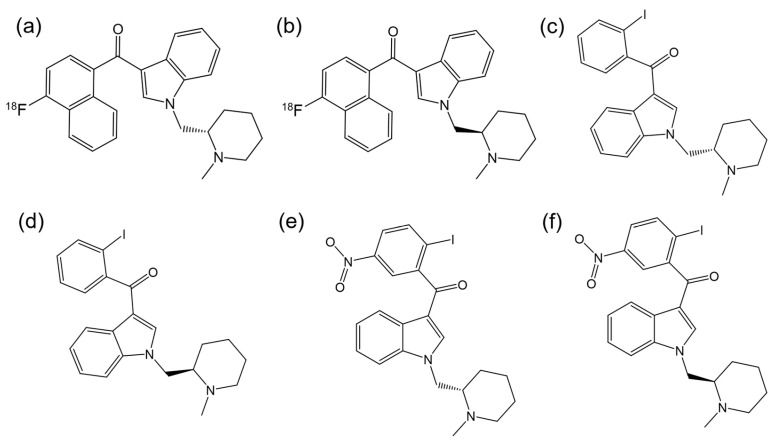
Chemical structures of (**a**) *S*-enantiomer of 3-(4-fluoronaphthoyl)-1-(*N*-methylpiperidin-2-ylmeth-yl)indole; (**b**) *R*-enantiomer of 3-(4-fluoronaphthoyl)-1-(*N*-methylpiperidin-2-ylmeth-yl)indole; (**c**) *S*-AM2233; (**d**) *R*-AM2233; (**e**) *S*-AM1241; (**f**) *R*-AM1241. (ChemDraw^®^ Professional 18.0.).

There are other notable examples of enantioselectivity involving the aminoalkylindole class of synthetic cannabinoids. For instance, Willis et al. [88] synthesized a novel series of aminoalkylindoles, and, through a structure–activity study, identified the racemic ligand with highest affinity for the CB1R, which was then labeled with [^18^F] (Figure 7a,b). They demonstrated that one enantiomer specifically binds to the CBR, while the inactive enantiomer may be useful for assessing the non-displaceable binding of the active enantiomer.

Deng et al. [89] synthesized a CB1Ragonist, AM2233 (Figure 7c,d). Experiments with radio-iodinated versions of the enantiomers and mouse hippocampal membranes showed that *R*-enantiomer had the highest affinity for CB1R. Little specific binding with the *S*-enantiomer and no specific binding with the *R*-enantiomer were observed in mouse brains lacking CB1R.

Bingham et al. [90] conducted cAMP inhibition assays to assess the antinociceptive efficacy of AM1241 (Figure 7e,f). This compound acts as an agonist for the human CB2R, with the *R*-enantiomer exhibiting greater affinity than the *S*-enantiomer. However, in pain models, the *S*-enantiomer was more effective as analgesic than either *R*-AM1241 or the racemate, with its in vivo effect mediated by CB2R. Rahn et al. [91] evaluated both racemic and enantiomeric forms of AM1241 for CB2R mediated antinociceptive effects. While *R*-AM1241 produced stronger antinociception at both higher and lower doses compared to *S*-AM1241 and racemic AM1241, it was found that these effects were not dependent on opioid receptors.

**Figure 8:**
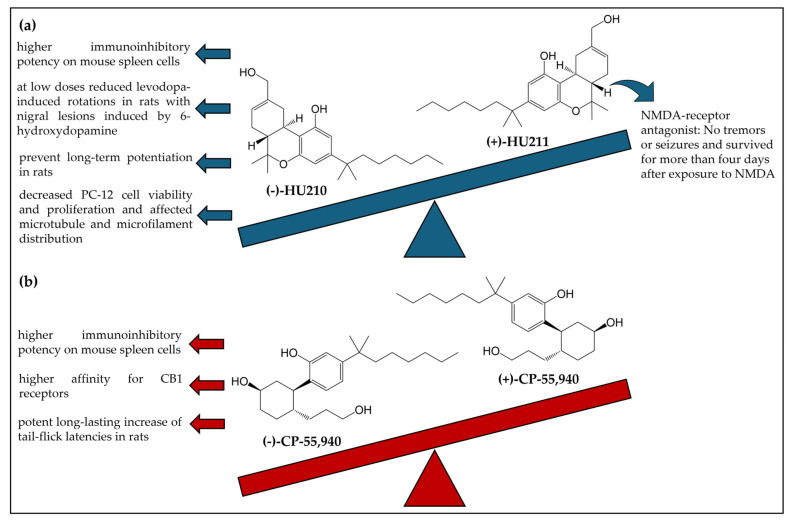
Chemical structures and enantiospecific effects of the enantiomers HU-210 (**a**) and CP-55,940 (**b**). CB1R: cannabinoid receptor 1; NMDA: *N*-methyl-D-aspartate.

Some enantioselectivity studies have also been performed with HU-210 [92,93,94,95,96] (Figure 8a) and CP-55,940 [54,93,97] (Figure 8b).

Generally, the studies showed that the (−)-enantiomer of both compounds displayed most of the effects while the (+)-enantiomers were less active or inactive. In only one study, the (+)-enantiomer of HU-210, designed in the literature as HU-211, displayed stronger effects. Feigenaum et al. [92] investigated the activity of HU-211. Mice treated with HU-211 showed no tremors or seizures and survived for more than four days after exposure to *N*-methyl-D-aspartate (NMDA). In contrast, HU-210 produced a sedative effect in mice but no significant impact on NMDA actions [92].

In recent years, carboxamide-type compounds have become more prevalent [13]. These substances were first developed by Pfizer and patented as potential analgesics; however, the patent only includes *S*-enantiomers due to the precursors (L-leucinamide, L-*tert*-leucine methyl ester and L-valinamide) [13,50,98]. The pharmacological activity of the *R*-enantiomers remains unknown [99]. Several studies evaluated the effect of chirality on the affinity and potency of various carboxamide-type synthetic cannabinoids to CB1R and CB2R [13,50,66,100,101,102,103,104,105]. The enantioselective results from these studies are summarized in Table 3.

The results showed that *S*-enantiomers exhibited greater potency and higher affinity for CB1R compared to *R*-enantiomers [13,50,66,101,102] with the exception of one study where the *R*-enantiomer showed greater activity [104]. In contrast, the effects on CB2R were more variable across studies. In studies by Antonides et al. [13] and Ametovsky et al. [101], *S*-enantiomers were also found to be more potent. Conversely, Baraldi et al. [100] and Stern et al. [104] reported stronger effects for the *R*-enantiomers. In a study by Doi et al. [50], three *R*-enantiomers and two *S*-enantiomers showed greater potency for CB2R.

Apart from the studies related to the interaction of carboxamide-type synthetic cannabinoids with CBRs, Brandon et al. [106] studied the structure–metabolism relationship and physicochemical parameters of twelve indazole and indole-3-carboxamide-type synthetic cannabinoids (AB-CHMINACA, AB-FUBINACA, AMB-FUBINACA, 5F-AMB-PINACA, AMB-CHMICA, AMB-4en-PICA, MDMB-4en-PINACA, 5F-MDMB-PINACA, 5F-MDMB-PICA, MDMB-4en-PICA, 4F-MDMB-BINACA, and MDMB-FUBINACA). In both cell models used, pooled cryopreserved human hepatocytes (pHHeps) and pooled human liver microsomes (pHLM), *R*-enantiomers were cleared at a slower rate than *S*-enantiomers. An exception was observed in the cell line pHLM for compounds containing an alkene tail group, including MDMB-4en-PICA, MDMB-4en-PINACA and AMB-4en-PICA.

### 3.2. Synthetic Cathinones

#### 3.2.1. PRISMA Search

**Figure 9:**
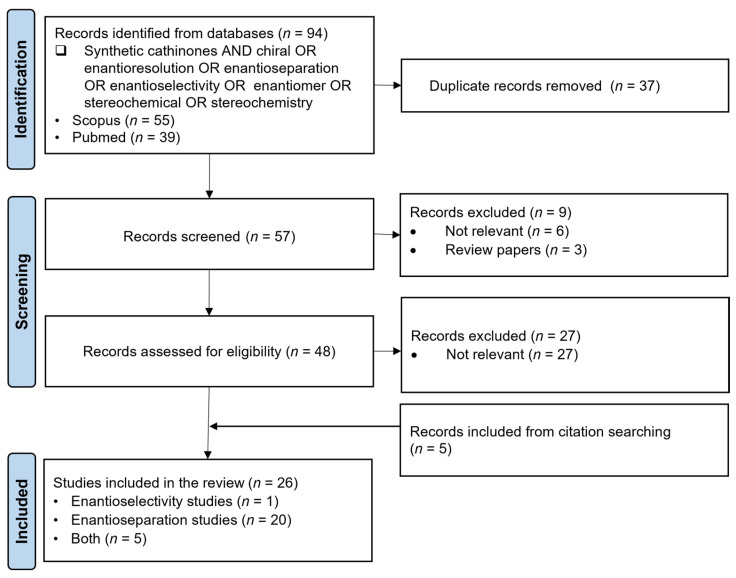
Flow diagram of the literature search based on PRISMA guidelines (*n* = number of scientific articles; time frame: 2022–February 2025; database: SCOPUS and Pubmed).

The PRISMA approach was conducted according to the flowchart presented in Figure 9. In the screening phase, some articles that seemed to meet the inclusion criteria had to be excluded since they were not relevant for the aims of this review. For instance, Lawtrakul and Toochinda [107] evaluated the potential of methylated β-cyclodextrin derivatives for the enantiorecognition of butylone. Moreover, Holowinski and Dybowski [108] studied the interactions of 3- and 4-chloromethcathinone with plasma proteins. In this report, the binding modes of the enantiomers of both synthetic cathinones in binding sites of human serum albumin (HSA) were analyzed using molecular docking and molecular dynamics studies [108]. Although both articles appear to be relevant for this research, they were excluded since they are theoretical analyses and therefore out of the scope of this review. Nonetheless, they highlight the potential of molecular docking as a predictive tool for both enantioselectivity and enantioseparation studies with synthetic cathinones. In total, 26 scientific articles were selected for further discussion, 1 focused on enantioselectivity study, 20 focused on enantioseparation and 1 addressing both enantioselectivity and enantioseparation.

#### 3.2.2. Enantioseparation (Update 2021–2025)

Enantioseparation methods for synthetic cathinones were previously reviewed by Silva et al. [29] up until 2018 and later updated by Almeida et al. [7], covering publications through to 2022. Table 4 provides an updated review of enantioseparation methods for this class of NPSs, spanning from 2022 to 2025.

A wide variety of methods can be used for enantioseparation, and they are generally classified into direct and indirect methods. In indirect methods, enantiomers are derivatized to form diastereomers, which are then separated using conventional separation techniques. In contrast, direct methods perform the separation in a chiral environment, using a chiral selector [65,129,130].

**Figure 10:**
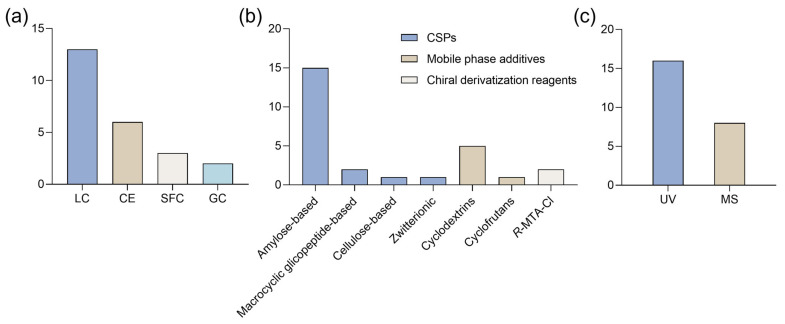
Bar charts regarding (**a**) type of technique, (**b**) chiral selectors/resolution agents, and (**c**) detection methods used for enantioseparation of synthetic cathinones. CE: capillary electrophoresis; CSPs: chiral stationary phases; GC: gas chromatography; LC: liquid chromatography; MS: mass spectrometry; *R*-MTPA-Cl: *R*-(−)-α-Methoxy-α-(trifluoromethyl)phenylacetyl chloride; SFC: super-critical fluid chromatography; UV: ultra-violet.

In this updated compilation, all studies, except for two that used an indirect method with GC (Figure 10a), employed direct enantioseparation methods based on LC, CE, or SFC techniques. In GC, indirect methods are more common. Despite its high efficiency and sensitivity, GC requires derivatization, and its high operating temperatures can lead to racemization or even decomposition of the analytes [57].

HPLC was the most frequently used technique for the enantioseparation of synthetic cathinones, showing a clear preference over other methods (Figure 10a). For example, Seibert et al. [119] tested an HPLC method for the enantioseparation of cathinone derivatives. Under the initial normal-phase conditions, 75 out of 80 compounds were successfully separated, while the remaining compounds achieved partial separation after optimization. Furthermore, using polar organic conditions, 63 compounds were enantioseparated with short retention times [119].

Beyond HPLC, three studies used SFC, while six used CE, a direct method based on capillary electromigration techniques that rely on electrophoretic phenomena to move the analyte [130]. In CE, a chiral selector, such as cyclodextrins, is typically added to the running buffer, forming a “pseudophase” that facilitates separation [131].

Most studies used polysaccharide-based CSPs, particularly amylose derivatives (Figure 10b). Additionally, one study applied an LC method with two macrocyclic glycopeptides-based CSPs, while another study used an SFC method with a zwitterionic CSP.

For five CE methods, cyclodextrin derivatives were added as chiral selectors in the mobile phase, while the remaining study used a cyclofructan derivative. In the two GC-MS methods, *(R*)-(−)-α-methoxy-α-(trifluoromethyl) phenylacetyl chloride (*R*-MTPA-Cl) was selected as the chiral derivatization reagent. Regarding detection methods, UV and MS detection were the most commonly used, with UV being preferred over MS (Figure 10c). However, both detection methods were also applied complementarily. For instance, in developing an enantioselective LC method to monitor MDPV in ecotoxicity assays, Pérez-Pereira et al. [115] tested amylose- and cellulose-based CSP, as well as various mobile phases, using ultra-fast liquid chromatography (UFLC) with a UV detector. The optimized conditions, including reverse-phase mode with an amylose CSP, were then applied to an LC-MS/MS system [115].

Regarding the type of samples analyzed, most of the synthetic cathinones were analyzed in powder form as hydrochloride salts. Nevertheless, Pérez-Alcaraz et al. [126] developed a CE-MS method for the enantiodetermination of MDPV in urine samples, while Langa et al. [125] applied a GC-MS method to analyze several chiral psychoactive substances, including synthetic cathinones, in effluents and river surface water samples. Additionally, Pérez-Pereira et al. [115] developed an LC-MS method to monitor the racemization of MDPV enantiomers in culture media from *Daphnia magna* ecotoxicity assays. These studies underscore the importance of enantioresolution methods for determining enantiomeric ratios in biological samples. Such analyses are crucial for assessing the prevalence of each enantiomer, enabling more accurate risk assessments, and detecting potential racemization to ensure the reliability of enantioselective results.

Additionally, it is important to infer that sample preparation is a critical step in analytical studies, particularly when working with chiral compounds, as improper handling can lead to racemization and degradation, ultimately compromising analytical results. In most of the studies, NPSs were analyzed in powder form and required no prior treatment beyond dissolution in a suitable solvent. However, some studies reported the need for additional sample preparation steps before analysis. Several extraction techniques such as liquid–liquid extraction, solid-phase extraction and microextraction are commonly applied. After extraction, samples are concentrated and reconstituted in an appropriate solvent that ensures compatibility with the analytical technique employed. It is crucial that these steps are optimized to maintain sample integrity and improve detection and quantification limits [132].

Compared to our previous reviews [7,29], which summarized enantioseparation studies on synthetic cathinones, the current findings confirm that the trend of using LC methods and polysaccharide-based CSPs remains strong.

When compared to the studies on synthetic cannabinoids, the literature on the enantioseparation of synthetic cathinones is significantly more advanced, with a greater number of studies available. This discrepancy can be attributed to several factors, the most obvious being the number of chiral compounds in each class of NPSs. While all synthetic cathinones are chiral, many groups of synthetic cannabinoids lack chirality, resulting in a lower number of chiral compounds available for study. Additionally, synthetic cathinones are small molecules with similar structures within each other. On the other hand, synthetic cannabinoids are bigger and more complex molecules that are divided into distinct groups with structural variabilities. Thus, while one enantioseparation method could be easily applied and adapted to several synthetic cathinones, the development of techniques for synthetic cannabinoids is more complex.

Some enantioselectivity studies on synthetic cannabinoids chose the “chiral approach” to synthesize enantiomerically pure compounds, rather than enantioseparating the racemates [50,66,67]. This may be due to the good availability of chiral precursors, such as amino acid derivatives, in their enantiomerically pure form. Nonetheless, these precursors are not always enantiomerically pure, which can result in enantiomeric mixtures with minor percentages of the opposite enantiomer. In such cases, as previously mentioned, enantioseparation methods could also be helpful for determining the enantiomeric purity of compounds.

#### 3.2.3. Enantioselectivity Studies (Update 2021-February of 2025)

Similarly to synthetic cannabinoids, synthetic cathinones have been extensively studied; however, research on enantioselectivity remains limited. Previously, our group reviewed studies on the enantioselective biological and toxicological activity of this NPS group up until 2018 [29]. We later published an updated review focusing on enantioselectivity research in synthetic cathinones [7]. The following section provides a literature review of enantioselectivity studies on synthetic cathinones conducted over the past three years (2021 to February of 2025).

**Figure 11:**
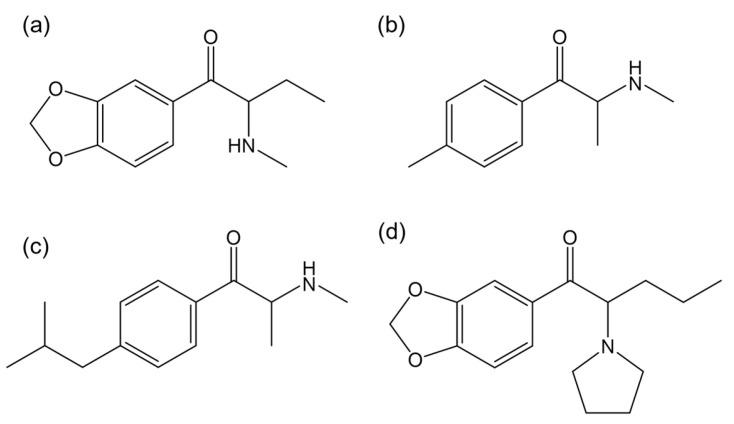
Chemical structures of the synthetic cathinones: (**a**) butylone; (**b**) mephedrone; (**c**) 4-isobutylmethcathinone; and (**d**) 3,4-methylenedioxypyrovalerone (MDPV). (ChemDraw^®^ Professional 18.0.).

Carvalho et al. [120] assessed the ecotoxicity of the enantiomers of butylone (Figure 11a) on *Daphnia magna*. In terms of body size, heart size, and area, *R*-butylone caused an increase in body size in juveniles at concentrations of 0.10 and 1 µg/L. In contrast, *S*-butylone led to a decrease in heart size and area in juveniles at 1 µg/L, suggesting that both enantiomers can selectively affect critical life stages of *Daphnia* in an enantioselective manner. Regarding swimming behavior, *S*-butylone increased the total swimming distance at 0.10 µg/L while *R*-butylone showed no effect [120].

A study by Czerwinska et al. [127] demonstrated that both *R*-(+)- and *S*-(−)-mephedrone presented similar kinetics; however, the *R*-(+)-enantiomer had a longer half-life, a higher maximum concentration, and a greater area under the curve. Therefore, the chiral nature and its associated enantiomeric purity must be considered when interpreting toxicological results. The chemical structure of mephedrone is shown in Figure 11b.

Paškan et al. [123] synthesized a new synthetic cathinone, 4-isobutylmethcathinone (Figure 11c). Subsequent in vitro studies in human neuroblastoma cells (SH-SY5Y), human microglial clone-3 cells (HMC-3), human liver cancer cells (hHep G2) and human cells derived from the urinary bladder (5637) cell lines showed no enantioselectivity in toxicity mechanisms. No enantioselectivity was observed in receptor binding studies either.

Pérez-Pereira et al. [115] evaluated the potential racemization of MDPV (Figure 11d) enantiomers in ecotoxicity assays. The results showed partial racemization of both enantiomers in chronic assays with *Daphnia magna*, with a higher extent of racemization for *S*-MDPV.

Almeida et al. [38] conducted an in vitro study with MDPV using the human colorectal adenocarcinoma (Caco-2) cell line. It was found that both enantiomers were highly permeable across the monolayer, and enantioselectivity in passage velocity was observed from the basolateral to apical direction. In another study by the same group [17], MDPV enantiomers exhibited cytotoxicity in a concentration-dependent manner. Additionally, no effects were observed on the expression of brain-derived neurotrophic factor and cyclin-dependent kinase 5 proteins in the SH-SY5Y cell line.

In the past three years, most studies on this topic have focused on the enantioseparation of synthetic cathinones. Although considerably less research has addressed the enantioselectivity of their biological and toxicological effects, it is important to highlight that this area of research is critically important. Given the prominence of synthetic cathinones among NPSs and their high abuse potential, failure to account for such differences between enantiomers can lead to underestimating the risk posed by specific enantiomers when assessing health hazards. Enantioselective toxicological data are crucial for accurately evaluating the potential harm associated with the use of these substances. This is particularly relevant given the prevalence of synthetic cathinones in recreational drug markets and their association with severe adverse effects, including neurotoxicity, cardiotoxicity, and acute behavioral disturbances [16]. Additionally, enantioselective data is critical for regulatory authorities aiming to implement effective control measures. Current regulations generally address racemic mixtures or the most common enantiomer; the lack of detailed toxicity data on individual enantiomers can lead to less accurate regulatory outcomes. Furthermore, the emergence of synthetic cathinones often involves minor structural modifications, including stereochemistry alterations, to evade legal restrictions [133]. Comprehensive enantioselective toxicological profiles would therefore support more robust and anticipatory regulatory frameworks.

**Figure 12:**
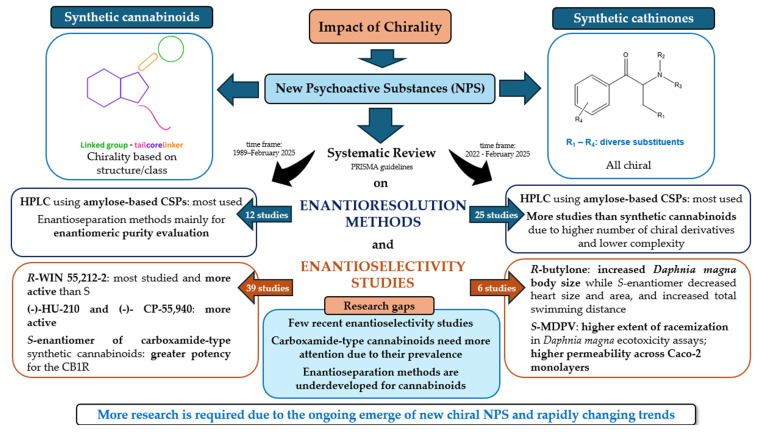
Diagram to illustrate the key points addressed in this review.

In summary, while advances in analytical methods for enantioseparation are crucial, they must be complemented by studies on the enantioselective toxicology of synthetic cathinones to ensure a holistic understanding of their health risks and to inform evidence-based regulatory responses. Figure 12 provides a summary of the key points covered in this review.

## 4. Conclusions

Although several enantioselectivity studies on synthetic cannabinoids have been conducted over time, the number of recent publications remains limited. Nonetheless, some studies have demonstrated that enantiomers can exhibit different properties. Continuing this line of research is crucial, as these differences in properties are closely linked to variations in pharmacological and toxicological outcomes. In particular, further investigation into the enantioselectivity of carboxamide-type synthetic cannabinoids is necessary due to their high prevalence on the NPS market. Additionally, our review found that enantioseparation studies involving synthetic cannabinoids are still relatively scarce.

Synthetic cathinones, on the other hand, have received more extensive recent attention regarding enantioselectivity and the development of enantioresolution methods compared to synthetic cannabinoids. Consequently, continued literature reviews in this area are essential to support future research and regulatory efforts.

The development of enantioresolution methods is closely linked to enantioselectivity studies, as it allows for the isolation of single enantiomers with high enantiomeric purity for enantioselective studies. Additionally, it enables the determination of whether a substance being sold is a racemate, a mixture of enantiomers, or a single enantiomer. Moreover, enantioselective analytical methods are highly valuable for the simultaneous analysis of both enantiomers in biological activity assays, allowing for the evaluation of enantioselective effects.

HPLC using CSPs, coupled with various detection methods, is the preferred technique for the enantioseparation of both substance groups. This systematic review, while following PRISMA guidelines, has limitations: it lacks a formal assessment of bias and may suffer from publication bias, potentially skewing the findings. Also, variations in study methods, samples, and conditions make direct comparisons difficult. Furthermore, the rapid emergence of NPSs means that the results might quickly become outdated.

This review highlights the clinical importance of understanding enantioselectivity in chiral narcotics or psychotropics, more specifically synthetic cannabinoids and synthetic cathinones. By presenting examples where one enantiomer exhibits markedly different toxicological profiles compared to its counterpart, the article emphasizes the need for enantiomer-specific research, monitoring, and harm reduction strategies. Recognizing these differences can support more accurate risk assessments, inform public health policies, and ultimately contribute to the better protection of individuals vulnerable to the toxic effects of these NPSs.

Future research should focus on the biological activity of individual enantiomers of NPSs to improve risk assessment and regulation, as their composition affects toxicity. Potential racemization could complicate results, highlighting the need for better methods to separate and identify NPS enantiomers in biological samples. Thus, more research and continuous literature review are necessary to better understand these evolving chiral NPSs.

## Figures and Tables

**Table 1:** Analytical methods for the enantioresolution of synthetic cannabinoids.

Analyte	Sample	Method	Analytical Conditions	Refs.
AB-CHMINACA, 5CI-AB-PINACA, AB-FUBINACA, ADB-FUBINACA, APP-FUBINACA, 5F-APP-PINACA	Whitish crystalline powders	HPLC-UV	Column: (*R*,*R*)-Whelk-O^®^ 1 and (*S*,*S*)-Whelk-O^®^1 Mobile phase: H_2_O/ACN/FA (55/45/0.1, *v*/*v*/*v*) Flow rate: 1 mL/min Temperature: 25 °C UV detection: 220 nm	[46]
AB-CHMINACA, 5CI-AB-PINACA, AB-FUBINACA, ADB-FUBINACA, APP-FUBINACA, 5F-APP-PINACA	Whitish crystalline powders	LC-MS/MS	Column: (*R*,*R*)-Whelk-O^®^ 1 Mobile phase: H_2_O/ACN/FA (55/45/0.1, *v*/*v*/*v*) Flow rate: 0.3 mL/min Temperature: 25 °C	
AMB-FUBINACA, AB-FUBINACA, 5F-MDMB-PINACA, AB-CHMINACA	Herbal material	HPLC-PDA	Column: Lux^®^ Amylose-1 Mobile phase: H_2_O/ACN (45/55 *v*/*v*); H_2_O/ACN (55/45 *v*/*v*) Flow rate: 0.2 mL/min Column: Lux^®^ i-Cellulose-5 Mobile phase: H_2_O/ACN (45/55 *v*/*v*); H_2_O/ACN (55/45 *v*/*v*) Flow rate: 0.2 mL/min	[13]
AMB-FUBINACA, AB-FUBINACA, 5F-MDMB-PINACA, AB-CHMINACA	Herbal material	UHPLC-PDA-MS/MS	Column: Lux^®^ Amylose-1 Mobile phase: H_2_O/ACN with 0.1% FA Flow rate: 0.2 mL/min Temperature: 30 °C	
MDMB-CHMICA	Herbal material	HPLC-UV	Column: CHIRALPAK^®^ IA-3 Mobile phase: Hex/2-PrOH (20/80 *v*/*v*) Flow rate: 0.6 mL/min Temperature: 40 °C UV detection: 290 nm	[47]
MDMB-CHMCZCA	Powder	HPLC-UV	Column: CHIRALPAK^®^ IA-3 Mobile phase: Hex/2-PrOH (20/80 *v*/*v*) Flow rate: 0.6 mL/min Temperature: 40 °C UV detection: 290 nm	[48]
5F-AB-PINACA, 5F-AMB	Herbal material	LC-HR-MS	Column: CHIRALPAK^®^ AZ-3R; CHIRALPAK^®^ AY-3R Mobile phase: H_2_O/ACN (50/50 *v*/*v*); H_2_O/ACN (55/45 *v*/*v*); H_2_O/ACN (60/40 *v*/*v)* Flow rate: 0.3 mL/min Temperature: 40 °C	[49]
AB-FUBINACA, APP-CHMINACA, 5F-EMB-PINACA, EMB-FUBINACA, MDMB-FUBICA	Oily residue, white solid, white powder	LC-MS	Column: CHIRALPAK^®^ AZ-3R Mobile phase: H_2_O/ACN (55/45 *v*/*v*) Flow rate: 0.3 mL/min Temperature: 40 °C	[50]
HU-210; HU-211; d,l-CP47, 497; d,l-3-epi CP47, 497; d,l-CP47, 497-C8; d,l-epi CP47, 497-C8 homolog; AKB-48; UR-144; AB-FUBINACA; AM-694; RCS-4; RCS-8; JWH-250; JWH-203; PB-22; JWH-019; JWH-073; JWH-200; AM-2201; JWH-122; JWH-081 JWH-018; JWH-018 2 -naphthyl-N- (1, 2-dimethylpropyl) isomer; JWH-018 2 -naphthyl-N- (1 ethylpropyl) isomer; JWH-016; JWH-018 2 -naphthyl-N- (1 methylbutyl) isomer; JWH-018 2 -naphthyl-N- (1,1-dimethylpropyl) isomer; JWH-018 2 -naphthyl-N- (2 methylbutyl) isomer; JWH-018 2 -naphthyl-N- (2, 2-dimethylpropyl) isomer; JWH-018 2 -naphthyl-N- (3 methylbutyl) isomer; JWH-018 2 -naphthyl isomer;	Seized drugs	UHPSFC-PDA-UV-MS	Column: Acquity UPC2^®^ Trefoil CEL1 Mobile phase: 2-PrOH/CO_2_ in gradient mode Flow rate: 1.25 mL/min Temperature: 55 °C UV detection: 215 or 273 nm	[51]
CCH, *trans*-CCH, CP-47497, CP-55940, HU-210, CBD, JWH-018, JWH-073, and JWH-250	Herbal material	SFC-ESI-MS	Column: ACQUITY UPC2^®^ Trefoil AMY1 Mobile phase: CO_2_/MeOH (90/10 *v*/*v*) in gradient mode Flow rate: 2.0 mL/min Temperature: 40 °C	[52]
JWH-018-(ω-1)−OH; AM2201-(ω-1)−OH	Urine	LC−MS/MS	Column: Phenomenex Lux^®^ Cellulose-3 Mobile phase: H_2_O/ACN (50/50, *v*/*v*); H_2_O/ACN (55/45, *v*/*v*); H_2_O/ACN (60/40, *v/v*) in gradient mode Flow rate: 0.3 mL/min Temperature: 40 °C	[53]
CP-55,940	Racemic cannabinoid	HPLC-UV	Column: CHIRALPAK^®^ AD Mobile phase: EtOH/Hex (15/85 *v*/*v*); EtOH/Hex (10/90 *v*/*v*); EtOH/Hex (5/95 *v*/*v*) Flow rate: 1.0 mL/min UV detection: 220 and 260 nm	[54]
Substituted 4-oxo-1,4-dihydroquinoline-3-carboxamide derivatives	Racemic cannabinoid	HPLC-UV	Column: CHIRALPAK^®^ AD/AS Mobile phase: Hex/2-PrOH (95–90/5–10 *v*/*v*) Flow rate: 1 mL/min Temperature: 20 °C UV detection: 220 nm	[55]
Substituted 4-oxo-1,4-dihydroquinoline-3-carboxamide derivatives	Racemic cannabinoid	HPLC-UV	Column: CHIRALPAK^®^ AD/AS Mobile phase: Hex/2-PrOH (95–60/5–40 *v*/*v*), Hex/1-PrOH (95–60/5–40 *v*/*v*) or Hex/EtOH (95–60/5–40 *v*/*v*) Flow rate: 1 mL/min Temperature: 25 °C UV detection: 217–222 nm	[56]

2-PrOH: propan-2-ol; ACN: acetonitrile; DAD: diode array detection; ESI: electrospray ionization; EtOH: ethanol; FA: formic acid; HR: high resolution; Hex: *n*-Hexane; HPLC: high-performance liquid chromatography; LC: liquid chromatography; MeOH: methanol; MS: mass spectrometry; PDA: photodiode array detector; SFC: super critical fluid chromatography; UHP: ultra high performance; UV: ultra-violet.

**Table 2:** Summary of the results from in vitro studies using *R*-(+)-WIN 55,212-2 and *S*-(−)-WIN 55,212-3.

Compound	In Vitro Model	Effect	Refs.
*R*	NG108-15 cell line	Inhibition of calcium current amplitude	[77]
	1321N1 astrocytoma and A-172 glioblastoma cell lines	Inhibition of the action of interleukin-1 (IL-1)	[79]
	Human glioma cells	Activation of the apoptotic mitochondrial pathway and DNA fragmentation	[72]
	Human embryonic kidney 293 (HEK293) cell line	Peroxisome proliferator-activated receptor-α (PPARα) as an important mediator in the effects of *R*-(+)-WIN 55,212-2 on the signaling cascade that regulates interferon-β (IFN-β) expression	[87]
	Primary cultures of rat cortical microglial cells	Inhibition of lipopolysaccharide-induced tumor necrosis factor (TNFα) release	[73]
*R* and *S*	Cultured primary sensory neurons	Release of calcitonin gene-related peptide (CGRP)	[78]
	Human umbilical vein endothelial (HUVEC) cell line	Inhibition of tissue factor (TF) protein expression	[80]

**Table 3:** Summary of enantioselective results from studies related to the interaction of carboxamide-type synthetic cannabinoids with cannabinoid receptors (CB1R and CB2R).

Compound	Higher Potency		Observations	Ref.
	CB1R	CB2R		
AB-FUBINACA 2-fluorobenzyl isomer	*S*	*R*	*R*-MDMB-FUBICA showed no CB1R activity	[50]
APP-CHMINACA	*S*	*S*		
EMB-FUBINACA	*S*	*R*		
5F-EMB-PINACA	*S*	*R*		
MDMB-FUBICA	*S*	*S*		
AMB-FUBINACA	*S*	*S*	Higher affinity to CB2R than CB1R	[13]
AB-FUBINACA	*S*	*S*		
AB-CHMINACA	*S*	*S*		
5F-MDMB-PINACA	*S*	*S*		
MDMB-PICA	*S*	*-*	Higher potency for *tert*-butyl (MDMB) derivatives	[66]
4F-MDMB-BINACA	*S*	*-*		
MDMB-4en-PINACA	*S*	*-*		
5F-MMB-PINACA	*S*	*-*		
MDMB-FUBINACA	*S*	*-*		
MMB-CHMICA	*S*	*-*		
MDMB-4en-PICA	*S*	*-*		
MMB-4n-PICA	*S*	*-*		
1-Pentyl-*N*-(1-phenylethyl)-1*H-*indole-3-carboxamide	*S*	*S*		[101]
1-(5-Fluoropentyl)-*N*-(1-phenylethyl)-1*H*-indole-3-carboxamide	*S*	*S*		
1-(Cyclohexylmethyl)-*N*-(1-phenylethyl)-1*H*-indole-3-carboxamide	*S*	*S*		
1-(4-Fluorobenzyl)-*N*-(1-phenylethyl)-1*H*-indole-3-carboxamide	*S*	*S*		
(1-(Cyclohexylmethyl)-7-methoxy-1*H*-indol-3-yl)(octahydro-2H-pyrido[1,2-a]pyrazin-2-yl)methanone	*S*	*-*	For synthetic cannabinoids with two chiral centers, the effects varied	[102]
(1-(Cyclohexylmethyl)-7-methoxy-1*H*-indol-3-yl)(hexahydropyrrolo[1,2-a]pyrazin-2(1*H*)-yl)methanone	*S*	*-*		
(1-(Cyclohexylmethyl)-7-methoxy-1*H*-indol-3-yl)(3,3-dimethylhexahydropyrrolo[1,2-a]pyrazin-2(1*H*)-yl)methanone	*S*	*-*		
(3-Cyclohexyl-2,3-dihydro-[1,4]oxazino[2,3,4-hi]indol-6-yl)(4-ethylpiperazin-1-yl)methanone	*R*	*-*		[103]
*N*3-(1-Phenylethyl)-4-oxo-1-pentyl-1,4-dihydroquinoline- 3-carboxamide	*-*	*R*		[104]
*N*3-(1-(2-Naphthyl)ethyl)-4-oxo-1-pentyl-1,4-dihydroquinoline- 3-carboxamide	*-*	*R*		
*N*3-(1-(1-Naphthyl)ethyl)-4-oxo-1-pentyl-1,4-dihydroquinoline- 3-carboxamide	*-*	*R*		
*N*-Cyclohexyl-3,7-dihydro-3-methyl-7-oxo-2*H*-[1,4]oxazino[2,3,4-ij]quinoline-6-carboxamide	*-*	*R*		[100]
*N*-Adamant-1-yl-3,7-dihydro-3-methyl-7-oxo-2*H*-[1,4]oxazino-[2,3,4-ij]quinoline-6-carboxamide	*-*	*R*		
*N*-Cycloheptyl-3,7-dihydro-3-ethyl-7-oxo-2*H*-[1,4]oxazino[2,3,4-ij]quinoline-6-carboxamide	*-*	*R*		
*N*-Adamant-1-yl-3,7-dihydro-3-ethyl-7-oxo-2*H*-[1,4]oxazino-[2,3,4-ij]quinoline-6-carboxamide	*-*	*R*		
*N*-Adamant-1-yl-3,7-dihydro-3-isopropyl-7-oxo-2*H*-[1,4]oxazino-[2,3,4-ij]quinoline-6-carboxamide	*-*	*R*		
*N*3-(1-(1,2,3,4-Tetrahydronaphthyl))-4-oxo-1-pentyl-1,4- dihydroquinoline-3-carboxamide		(+)		[105]
*N*3-(1-(1-Adamantyl)ethyl)-4-oxo-1-pentyl-1,4-dihydroquinoline-3-carboxamide		(−)		
*N*3-(1-(1-Adamantyl)ethyl)-6-chloro-4-oxo-1-pentyl-1,4- dihydroquinoline-3-carboxamide		(+)		
*N*3-(1-(1-Adamantyl)ethyl)-7-chloro-4-oxo-1-pentyl-1,4- dihydroquinoline-3-carboxamide		(−)		

**Table 4:** Analytical methods for enantioseparation of synthetic cathinones.

Analyte	Sample	Method	Analytical Conditions	Refs.
bk-IVP, 5-PPDi, PV8, PV9, PV10, Naphyrone, TH-PVP, 5-DBFPV, MDPV, 3,4-MD-PHP	Hydrochloride salts	SFC-DAD	Column: Lux^®^ i-Amylose-3 (AMY-Cl) 3 μm Mobile phase: Various CO_2_/Organic solvent/Addictive (*v*/*v*/*v*) Flow rate: 2 mL/min Temperature: 40 °C UV detection: 220, 254 or 280 nm	[109]
3-MMC, 4-MMC, 3,4-DMMC, 4-MEC, MDMC	Hydrochloride salts	CE-UV	BGE: 20 mM phosphate (pH 2.5) Chiral selector: S-β-CD-CA Temperature: 25 °C	[110]
2-Fluormethcathinone, 3-Fluormethcathinone, 4-Fluormethcathinone	Hydrochloride salts	CE-DAD	BGE: 20 mM monobasic sodium phosphate (pH 2.5) Chiral selector: 14.36 mM CM-β-CD and 0.75% ChCl-EG (*v*/*v*) Temperature: RT UV detection: 210 nm	[111]
4-F-PV8, α-PVP, 4-Cl-PVP, 4-F-PVP, 4-MeO-α-PVP, 4-MPrC, α-PPP, M-PPP, α-PiHP, Naphyrone, TH-PVP, PV9, PV10, 5-DBFPV, 4-M-PHP, 3,4-MD-PHP, 4-CBC, 4-CIC, 4-CDC	Hydrochloride salts	SFC-DAD	Column: AZYP TeicoShell 2.7 μm and AZYP NicoShell 2.7 μm Mobile phase: Various CO_2_/Organic solvent/Addictive (*v*/*v*/*v*) Flow rate: 2 mL/min Temperature: 40 °C UV detection: 220, 254, 280 nm	[112]
3-MMC, 3,4-DMMC, 4-MEC, MDMC, MDPV	Hydrochloride salts	CE-DAD	BGE: 20 mM monobasic sodium phosphate (pH 2.5) Chiral selector: 1 mM SCF-7 Temperature: 25 °C UV detection: 210 nm	[113]
*N*-Cyclohexylmethylone	Crystalline powder	HPLC-UV	Column: Lux^®^ i-Amylose-3 Mobile phase: Hex/2-PrOH/DEA (95/5/0.1 *v*/*v*/*v*) Flow rate: 1.0 mL/min Temperature: 25 °C UV detection: 254 nm	[114]
MDPV	Hydrochloride salts	UFLC-UV	Column: Lux^®^ 3 μm—Cellulose-2 Mobile phase: Hex/EtOH/DEA (99/1/0.1 or 97/3/0.1 *v*/*v*/*v*) or Hex/2-PrOH (99:1, 98:2 or 97:3), 5 or 20 mM NH_4_OAc in UPW (pH 8.5 or 8.7)/EtOH or ACN (from 75/25 to 50/50) Flow rate: 0.3–1 mL/min Temperature: RT-35 °C UV detection: 315 nm	[115]
MDPV	Hydrochloride salts	UFLC-UV	Column: Daicel^®^ 3 μm—CHIRALPAK^®^ IF-3 Mobile phase: 5 mM NH_4_HCO_3_ in UPW (pH 8.8)/ACN (10/90 *v*/*v*) Flow rate: 0.3–1 mL/min Temperature: 30 °C UV detection: 315 nm	
MDPV	Culture media spiked	LC-MS/MS	Column: Daicel^®^ 3 μm—CHIRALPAK^®^ IF-3 Mobile phase: 5 mM NH_4_HCO_3_ in UPW (pH 8.8)/ACN (10/90 *v*/*v*) Flow rate: 0.3 mL/min Temperature: 30 °C	
2-Amino-3,4-dihydronaphthalene-1(2*H*)-one, 2-Amino-6-fluoro-3,4-dihydronaphthalene-1(2*H*)-one, 2-Amino-6-chloro-3,4-dihydronaphthalene-1(2*H*)-one, 2-Amino-6-bromo-3,4-dihydronaphthalene-1(2*H*)-one, 2-Amino-6-methyl-3,4-dihydronaphthalene-1(2*H*)-one, 2-Amino-6-methoxy-3,4-dihydronaphthalene-1(2*H*)-one	Hydrochloride salts	HPLC-UV	Column: Chiral ART Amylose-C Mobile phase: Hep/2-PrOH/HCOOH/IPA (90/10/0.1/0.1 *v*/*v*/*v*/*v*) Flow rate: 1.0 mL/min UV detection: 252 nm	[116]
Buphedrone, Butylone, 3,4-DMMC, 3-MMC	Solid	GC-MS	Column: SH-Rxi-5 ms Derivatization reagent: *R*-MTPA-Cl Flow rate: 1.0 mL/min	[117]
Ephylone, 4F-NEB, Pyrovalerone, Clephedrone, Pentylone, Mephedrone, Methedrone	Hydrochloride salts	SFC-ESI-MS	Column: CHIRALPAK^®^ ZWIX (+) Mobile phase: CO_2_/MeOH with 6.25 mM formic acid and 12.5 mM ammonium formate in gradient mode Flow rate: 1.0 mL/min Temperature: 35 °C	[118]
3-MMC, 4-MMC, 2-CMC, 3-CMC, 4-CMC, 2-FMC, 3-FMC, 4-FMC, 3-BMC, 4-BMC, 2-EMC, 3-EMC, 4-EMC, 3-MeOMC, 4-MeOMC, 2,4-DMMC, 3,4-DMMC, Buphedrone, Pentedrone, Mexedrone, 4-Methylbuphedrone, Ethcathinone, 3-MEC, 4-MEC, 3-CEC, 4-CEC, 3-FEC, 2-EEC, 3-EEC, 4-EEC, N-Ethylbuphedrone, *N*-Ethylpentedrone, *N*-Ethylhexedrone, Amfepramone, DL-4662, NiPP, 4-MPC, 4-FNPP, 4-ClC, 4-CBC, 4-CDC, NDH, 4-MBC, MDC, 2,3-MDMC, Methylone, 2-AIMP, Dimethylone, Butylone, Ethylone, Ephylone, 5-ME, MDPT, BMDP, BMDB, bk-iVP, 5-PPDi, 5-BPDi, α-PPP, M-PPP, α-PVP, 4-F-PVP, 4-Cl-PVP, 4-MPrC, 4-MeO-α-PVP, 4-MPHP, 4-F-PHP, PV8, 4-F-PV8, PV9, PV10, α-PIHP, α-PCYP, Naphyrone, 5-DBFPV, TH-PVP, BOH-PHP, MDPV, 3,4-MD-PHP, MDPEP	Hydrochloride salts	HPLC-UV	Column: Lux^®^ i-Amylose-3 Mobile phase: Hex/2-PrOH/DEA (95/5/0.1; 97/3/0.1; 99/1/0.1 *v*/*v*/*v*) Hex/EtOH/DEA (95/5/0.1 *v*/*v*/*v*) ACN/2-PrOH/DEA (95/5/0.1 *v*/*v*/*v*) Flow rate: 1.0 mL/min Temperature: 25 ± 1 ° C UV detection: 254 nm	[119]
Butylone	Hydrochloride salts	HPLC-DAD	Column: Homemade 3,5-dimethylphenylcarbamate amylose column coated with APS-Nucleosil (500 Å, 7 µm particle size, 20%, *w*/*w*) Mobile phase: Hex/EtOH/DEA (70/30/0.1 *v*/*v*/*v*) Flow rate: 1.5 mL/min Temperature: RT DAD: set at 230 nm	[120]
Pentylone, 4-MEC, Methylone, MDPBP, MDPV, Naphyrone	Hydrochloride salts	CE-UV	BGE: ammonium formate buffer (pH 3.1) 50 mM ionic strength with TBAC Chiral selector: β-CD UV detection: 214 nm	[21]
Pentylone, Bk-DMBDB, MDPV, Methylone, Pentedrone, 4-CEC, 3-CMC, 4-Cl-α-PVP, Buphedrone, Ethcathinone, 4-CMC, 4-FMC, 4-MEC, 4F-PHP	Hydrochloride salts in powder form	HPLC-UV	Column: CHIRALPAK^®^ AS-H and Lux Amylose-1^®^ Mobile phase: Hex/2-PrOH/TEA (97/3/0.1 *v*/*v*/*v*); Hex/EtOH/TEA (97/3/0.1 *v*/*v*/*v*); Hex/EtOH/DEA (97/3/0.1 *v*/*v*/*v*) Flow rate: 0.5 mL/min UV detection: 254 nm	[36]
2-(Methylamino)-1-(3-(trifluoromethyl)phenyl)propane-1-one and 2-(Methylamino)-1-(3-(trifluoromethyl)phenyl)pentane-1-one	Hydrochloride salt	HPLC-DAD	Column: ChiralART^®^ Amylose-SA Mobile phase: Hep/2-PrOH/DEA (95/5/0.1, *v*/*v*/*v*) Flow rate: 10 or 15 mL/min Temperature: 15 or 22 °C	[121]
MDPV	Hydrochloride salts	HPLC-UV	Column: Homemade column of tris-3,5-dimethylphenylcarbamate amylose coated onto aminopropylsilyl Nucleosyl (500 Å, 7 µm, 20%, *w*/*w*) Mobile phase: Hex/EtOH/DEA, (97/3/0.1 *v*/*v*/*v)* Flow rate: 1.5 mL/min UV detection: 254 nm	[17]
α-PVP, MDPV, 4-F-PHP, 4-Cl-α-PVP	Hydrochloride salts	LC/MS-MS	Column: CHIRALPAK^®^ IF Mobile phase: 5 mM ammonium bicarbonate buffer (pH 8.8)/ACN in gradient mode Flow rate: 0.5 mL/min Temperature: 30 °C	[122]
4-Isobutylmethcathinone	Hydrochloride salts	HPLC-UV	Column: CHIRALPAK^®^ IA Mobile phase: Hep/2-PrOH/DEA (90/10/0.1 *v*/*v*/*v*) Flow rate: 15 mL/min Temperature: 15 °C UV detection: 254 nm	[123]
4-FMC, 4-CMC, 4-Fluoropentedrone, 4-CDC, Buphedrone, *N*-Ethylheptdrone, *N*-Butylhexedrone, *N*-ethylbuthylone, *N*-butylpentilone, 4-BMC, 4-F-*N*-isopropylpentedrone, 4-CEC, Isopentedrone, 4-Methyl-*N*-Ethylpentedrone, *N*-Ethylhexedrone, *N*-Propylpentedrone, 4-Methylpentedrone, BMDP, NEB	Hydrochloride salts	CE-UV	BGE: 25 mM sodium dihydrogen phosphate (pH 7.0; 5.0; 2.5) Chiral selector: 2, 5, 7, and 10 mM of CD (Sugammadex, Subetadex and Sualphadex) Temperature: 25 °C UV detection: 200 and 254 nm	[124]
MDMA, Buphedrone, Butylone, 3,4-DMMC, 3-Methylmethcathinone	Estuarine water samples or effluent samples	GC–MS	Column: Zebron capillary column (achiral) Derivatization reagent: *R*-MTPA-Cl Flow rate: 1.0 mL/min	[125]
MDPV	Urine	SPE-CE-MS	BGE: 10 mM ammonium acetate (pH 7.0) Chiral selector: 0.5% (*m*/*v*) of sulphated-α-CD Temperature: 25 °C	[126]
Mephedrone	Hydrochloride salts	LC-MS/MS	Column: Lux^®^ Amylose-1 Mobile phase: EtOH:MeOH:DEA (20/80/0.1 *v*/*v*/*v*) Flow rate: 0.1 mL/min Temperature: RT	[127]
Methylone, Ethylone	Hydrochloride salts	LC-MS/MS	Column: Lux^®^ AMP polysaccharide-based chiral Mobile phase: H_2_O/MeOH in gradient mode Flow rate: 0.48 mL/min Temperature: 30 °C	[128]

2-PrOH: propan-2-ol; 4F-NEB: 4-Fluoro-*N-*ethylbuphedrone; β-CD: β-Cyclodextrin; ACN: acetonitrile; BGE: background electrolyte; CA: citric acid; CD: cyclodextrin; CE: capillary electrophoresis; ChCl: choline chloride; CM: carboxymethyl; DAD: diode array detection; DEA: diethylamine; EG: ethylene glycol; ESI: electrospray ionization; EtOH: ethanol; GC: gas chromatography; Hep: heptane; Hex: *n*-Hexane; HPLC: high-performance liquid chromatography; IPA: isopropylamine; LC: liquid chromatography; MeOH: methanol; MS: mass spectrometry; *R*-MTPA-Cl: *R*-(−)-α-Methoxy-α-(trifluoromethyl)phenylacetyl chloride; RT: room temperature; S: sulfated; SCF: sulfated cyclofructan; SFC: super-critical fluid chromatography; TBAC: tetrabutylammonium chloride; TEA: triethylamine; UFLC: ultra-fast liquid chromatography; UV: ultra-violet.

## Data Availability

Not applicable.
